# Effects of Dietary *Bacillus amyloliquefaciens* TL106 on Growth Performance, Antioxidant Capacity, Immune Function and Intestinal Microbiota in Danzhou Chickens

**DOI:** 10.3390/vetsci13090883

**Published:** 2026-08-28

**Authors:** Xinlei Wang, Peijie Du, Chong Xi, Yunhe Cao, Chunlin Wang

**Affiliations:** 1 College of Animal Science and Technology, China Agricultural University, Beijing 100193, China; wangxinlei0616@163.com (X.W.); dupeijie2002@163.com (P.D.); nd2026123@163.com (C.X.); 2 Sanya Institute of China Agricultural University, Sanya 572025, China; 3 State Key Laboratory of Animal Nutrition and Feeding, China Agricultural University, Beijing 100193, China

**Keywords:** *Bacillus amyloliquefaciens*, Danzhou chicken, growth performance, intestinal microbiota

## Abstract

The study aimed to investigate the effects of *Bacillus amyloliquefaciens* TL106 (TL106) on growth performance, antioxidant capacity, immune function, and intestinal microbiota in Danzhou chickens. A total of 384 healthy female Danzhou chicks were randomly assigned to a control group (CON) and three experimental groups (Ba1, Ba2, Ba3), receiving basal diets supplemented with TL106 at 5 × 10^7^, 5 × 10^8^, and 5 × 10^9^ CFU/kg, respectively, for 55 d (divided into early [1–35 d] and later [36–55 d] phases). While no significant growth improvement was observed in the early phase, average daily feed intake (ADFI) and final body weight (BW) increased, and the feed-to-gain ratio (F/G) was significantly reduced (*p* < 0.05) during the later phase in Ba2 and Ba3, especially in Ba3. At d 55, TL106 supplementation significantly elevated serum IgM content (*p* < 0.001) and total superoxide dismutase (T-SOD) activity (*p* < 0.05), reduced malondialdehyde (MDA) content (*p* < 0.01), and enhanced albumin (ALB) levels (*p* < 0.05). The Ba3 group also improved the villus height-to-crypt depth ratios (VH/CD) of the ileum and duodenum (*p* < 0.05). Microbial analysis indicated that abundances of Bacteroidota and Firmicutes increased while Proteobacteria and butyrate-producing Faecalibacterium were enriched in the TL106 groups compared to CON. Cecal butyrate concentrations rose dose-dependently (*p* < 0.05), with most of the key parameters—including final BW, ADFI, F/G, serum ALB, IgM, T-SOD, GSH-Px, MDA (d 55), duodenal and ileal VH/CD, and cecal butyric, propionic, and valeric acids—showing significant quadratic responses (*p* < 0.05). The results demonstrated that TL106 in diets enhanced growth performance and intestinal health in Danzhou chickens by modulating immunity, antioxidant responses, and microbiota, with 5.0 × 10^8^ CFU/kg appearing to be the most effective dose among those tested under the conditions of this study.

## Simple Summary

*Bacillus amyloliquefaciens*, a Gram-positive, facultative anaerobic bacterium, has recently emerged as a novel, safe, and environmentally friendly feed additive in the field of livestock and poultry feeding. This experiment found that adding *Bacillus amyloliquefaciens* TL106 to the diet was associated with enhanced immune response, alleviated oxidative damage, maintained intestinal morphology, optimized intestinal microbiota structure, and synergistically improved growth performance in Danzhou chickens to a certain extent. The effects showed dose-dependent and quadratic relationships. Overall, the study provided a valuable reference for the precise application of *Bacillus amyloliquefaciens* TL106 in Danzhou chicken production.

## 1. Introduction

Probiotics, as environmentally friendly feed additives, optimize nutrient absorption efficiency and improve livestock and poultry performance and product quality by regulating gut microbiota structure and activating immune responses [1]. Probiotics have become an important alternative to antibiotics due to their non-toxic properties and safety advantages. Among numerous probiotic preparations, *Bacillus amyloliquefaciens* has become one of the highly regarded probiotic species due to its unique biological characteristics and functional benefits, and is widely applied in animal husbandry [2,3].

At present, Danzhou chicken has been included in the List of Chinese Livestock and Poultry Genetic Resources Varieties. Being a native breed in Hainan, Danzhou chicken holds a significant position in local animal husbandry due to its heat stress resistance, robust disease resistance, as well as delicious meat [4]. With the increasing demand for high-quality poultry products, improving the productivity and health status of Danzhou chickens has become a key pursuit for the development of the industry.

*Bacillus amyloliquefaciens*, as a bacterium widely present in natural environments, can produce various bioactive substances, such as enzymes, antibiotics, and prebiotics, showing great potential in promoting the digestion and absorption of animal nutrients, regulating the body’s antioxidant system, enhancing immune response, and improving the structure of gut microbiota [5,6,7]. Notably, the complete genome sequencing of *Bacillus amyloliquefaciens* TL106 (TL106) has revealed a rich repertoire of carbohydrate-active enzymes (CAZymes), including cellulases, xylanases, and β-glucanases, which underlie its strong fiber-degrading capacity and potential probiotic functions [8]. However, there is currently a lack of research on the impacts of *Bacillus amyloliquefaciens* on Danzhou chickens, particularly regarding the application of the specific strain TL106 in these chickens. It is well known that Danzhou chickens in large-scale breeding are susceptible to intestinal diseases due to the consistently high temperature and humidity in Hainan, and the use of antibiotics is relatively high in traditional farms. Therefore, conducting in-depth research on the effects of TL106 on growth performance, antioxidant capacity, immune function, and gut microbiota in Danzhou chicken not only aims to address its industrial pain points through the intervention of Bacillus, but also has the potential to provide innovative micro-ecological regulation strategies for the efficient and healthy production of Danzhou chicken. This research is anticipated to provide a theoretical foundation and practical guidance for the development of safe, green, and sustainable poultry farming technologies.

## 2. Materials and Methods

### 2.1. The Strain

The lyophilized powder of *Bacillus amyloliquefaciens* TL106 used in this experiment was prepared by fermentation in a 30-L fermenter and subsequent freeze-drying, with no carrier material added. The live spore number was 1.0 × 10^10^ CFU/g. The viability was determined by plate counting; purity was assessed by colony morphology and Gram staining, with no contamination detected (viable count ≥ 1.0 × 10^10^ CFU/g, purity ≥ 95%).

### 2.2. Animals and Diets

Danzhou chickens in the experiment were purchased from Hainan Rekeyuan Ecological Breeding Co., Ltd., Danzhou, China. A total of 384 one-day-old black chickens were randomly divided into four groups, with six replicates in each group, and 16 chickens per replicate. The experimental treatments included four groups. The control group (CON) was fed a basic diet, while the Ba1, Ba2, and Ba3 groups were supplemented with 5.0 × 10^7^, 5.0 × 10^8^, and 5.0 × 10^9^ CFU/kg of TL106, respectively. The experiment was conducted over 55 days and was divided into an early phase (1–35 d) and a late phase (36–55 d). The diets for Danzhou chicken were formulated according to the nutrient requirements for slow-growing yellow chickens as specified in Nutrient Requirements for Yellow Chickens (NY/T 3645-2020, China). The composition and nutrient levels of the diet were detailed in Table 1.

### 2.3. Animal Management

The experiment was carried out at Tropical Crop Genetic Resources Institute, Chinese Academy of Tropical Agricultural Sciences. One-day-old Danzhou chickens were inoculated with Marek’s vaccine. The Newcastle vaccine and the infectious bronchitis vaccine were administered at d 4 and d 21, respectively, followed by the infectious bursal disease inactivated vaccine at d 14 and d 28 successively. In the initial two weeks, continuous 24-h lighting was adopted to ensure that the chickens could acclimate to the environment and find the feed and water sources. Subsequently, a lighting schedule of 23 h of light and 1 h of darkness was implemented daily. All chickens had ad libitum access to feed and water during the trial period, and ventilation was maintained as needed. The initial room temperature was set at 38 °C and gradually reduced by 2 °C each week until 24 °C at d 21. Throughout the trial, the relative humidity was strictly maintained within the range of 60% to 80%. The rearing of Danzhou chickens followed the guidelines established under commercial practices.

## 3. Sample and Measurement

### 3.1. Growth Performance

At 8:00 on d 1, 35, and 55 of the experiment, the remnant feed was collected by cage and weighed. At 22:00, the birds were weighed. Twelve hours before the end of each stage, the diet was stopped to keep the chickens in a fasting state. Subsequently, the body weight (BW) of the chickens in each cage was measured. Ultimately, average daily gain (ADG), average daily feed intake (ADFI), and feed-to-gain ratio (F/G) were calculated.

### 3.2. Serum Biochemical Indices

On d 35 and 55, six chickens were randomly selected from each treatment, and blood samples were collected from the jugular vein. The samples were allowed to coagulate at room temperature, and then centrifuged at 3000 rpm for 10 min to harvest the serum. The serum was immediately placed in an ice box and stored at −80 °C for subsequent determination. The enzyme-linked immunosorbent assay (ELISA) kit and the microplate reader were used to determine the serum cytokines and oxidative stress indicators, including total protein (TP), albumin (ALB), immunoglobulin A (IgA), immunoglobulin G (IgG), immunoglobulin M (IgM), total superoxide dismutase (T-SOD), glutathione peroxidase (GSH-Px), malondialdehyde (MDA), catalase (CAT) content. Total antioxidant capacity (T-AOC) and the content of tumor necrosis factor-α (TNF-α), and the inflammatory indicators interleukin-6 (IL-6) and interleukin-10 (IL-10) were also determined. The ELISA kits were purchased from Nanjing Jiancheng Bioengineering Institute (Nanjing, China).

### 3.3. Intestinal Tissue Morphology

Chickens were randomly selected from each treatment for intestinal tissue collection. The samples fixed with paraformaldehyde were placed in embedding boxes and neatly put into a dehydrator for dehydration. Finally, all samples were transferred to an embedding machine, and then the embedded tissues were cut into 4-µm sections and placed on a glass slide. After baking at 60 °C for 5 h on a baking device, hematoxylin-eosin staining was carried out. For observation, three discontinuous sections were made from each sample. A microscope (Olympus BX 51, Olympus Corporation, Tokyo, Japan) was used for image acquisition and the measurement of villus height (VH) and crypt depth (CD). Additionally, the ratio of villus height to crypt depth (VH/CD) was calculated.

### 3.4. Short-Chain Fatty Acids (SCFA) Levels

Chickens were selected randomly for slaughter to collect cecal chyme. The cecal content was retrieved, placed into a 5-mL cryotube, and promptly frozen in liquid nitrogen. The cryopreserved samples were subsequently transferred to a −80 °C freezer for storage pending further analysis. The levels of SCFA in the filtered sample solution were determined using a high-performance ion chromatograph (ICS-3000, Thermo Fisher Scientific, Waltham, MA, USA). The concentrations of various SCFA, such as acetic acid, propionic acid, and butyric acid, were accurately analyzed.

### 3.5. Intestinal Microbiota Analysis

The 16S rRNA gene sequence analysis technology was employed to analyze the microbial communities present in the cecal chyme of each group. The DNA in the cecal chyme was extracted using a DNA extraction kit (Omega Bio-tek, Norcross, GA, USA). Universal bacterial primers 338F (5′-barcode ACTCCTACGGG AGGCAGCAG-3′) and 806R (5′-GGACTACHVGGGTWTCTAAT-3′) were used for polymerase chain reaction (PCR) amplification. Sequencing was carried out on the Illumina HiSeq sequencing platform. Bioinformatics analysis of the microbiota was performed by Shanghai Personal Cloud Platform (https://www.genescloud.cn/ (accessed on 1 December 2024)). Alpha diversity indices, including Chao1 and Shannon, were calculated based on operational taxonomic unit (OTU) information using QIIME2. The similarity of the microbial communities in different samples was determined by Principal coordinate analysis (PCoA) based on the Bray–Curtis index. Linear discriminant analysis (LDA) effect size (LEfSe) was used to identify the abundance (LDA > 2, *p* < 0.05) of bacterial taxa (from phylum to genus) among groups.

## 4. Statistical Analysis

The general linear model (GLM) of SPSS 27.0 was used. One-way analysis of variance (ANOVA) was conducted to assess significant differences among groups, and the Duncan method was employed for multiple comparisons of the means. The analyzed data were presented in the form of the mean and the standard error of the mean (SEM). A quadratic regression model was employed to evaluate the addition level of TL106. When *p* < 0.05, it was considered a statistically significant difference, and when 0.05 ≤ *p* < 0.1, it indicated a trend of significant difference. In Table 2, Table 3, Table 4, Table 5 and Table 6, “Interblock” represents the overall treatment effect (between-group differences) assessed by one-way ANOVA. Linear and quadratic *p*-values were derived from orthogonal polynomial contrasts using dose-level coding (0, 1, 2, 3 for the four supplementation levels: 0, 5 × 10^7^, 5 × 10^8^, and 5 × 10^9^ CFU/kg).

## 5. Results

### 5.1. Growth Performance

The growth performance results of Danzhou chickens are presented in Table 2. In the later stage, the ADFI of TL106 groups was significantly higher than that of CON (*p* < 0.05), and F/G in the Ba2 and Ba3 groups was significantly lower than that in the Ba1 and CON groups (*p* < 0.05). There was a linear and quadratic curve relationship for ADFI (*p* < 0.05), and a quadratic curve for F/G (*p* < 0.01). At d 55, the BW of TL106 groups was higher than that of CON, and there were linear and quadratic relationships (*p* < 0.05). During the whole stage, ADFI of TL106 groups was higher than that of CON (*p* < 0.05). F/G of TL106 groups was significantly lower than that of CON (*p* < 0.05). There were linear and quadratic relationships for ADFI (*p* < 0.05), and F/G showed a quadratic curve decrease (*p* < 0.05). Overall, dietary TL106 supplementation improved growth performance in Danzhou chickens, primarily during the later phase (36–55 d). 5.0 × 10^8^ CFU/kg was the dose selected among the supplementation levels tested in this experiment that appeared to be more appropriate relative to the other doses under the conditions of this study.

### 5.2. Cytokine and Immunity Indices

At d 35, the IL-6 level in group Ba3 was significantly lower than that in CON (*p* < 0.05). The TNF-α level in Ba3 was significantly lower than that in other groups (*p* < 0.05). The results are shown in Table 3. There was a quadratic curve relationship for IL-6 and both linear and quadratic relationships for TNF-α (*p* < 0.05). At d 55, the ALB levels in the Ba2 and Ba3 groups were significantly higher than those in CON and Ba1 (*p* < 0.05). The IgM level in Ba3 was significantly higher than that in the others (*p* < 0.001). The TNF-α levels in Ba3 and Ba2 were significantly lower than those in CON and Ba1 (*p* < 0.05). There was a quadratic curve relationship for ALB, linear and quadratic relationships for IgM, and a linear relationship for TNF-α (*p* < 0.05).

### 5.3. Antioxidant Capacity

At 35 d, dietary TL106 significantly increased the activity of T-SOD, and the Ba2 and Ba3 groups significantly reduced MDA content (*p* < 0.05). Table 4 presents the results. There was a quadratic curve relationship for T-SOD, and a linear relationship for MDA (*p* < 0.05). At d 55, the activity of GSH-PX in Ba2 was significantly higher than that of CON (*p* < 0.05). The T-SOD activity in Ba3 was significantly higher than that in other groups (*p* < 0.01). The MDA content in Ba2 and Ba3 was lower than that in CON and Ba1 (*p* < 0.05). There was a quadratic curve relationship for GSH-PX (*p* < 0.05), a highly significant quadratic for T-SOD (*p* < 0.001), and a quadratic for MDA (*p* < 0.01). Therefore, adding TL106 can increase T-SOD activity and reduce MDA content in serum, thus enhancing serum antioxidant capacity in Danzhou chickens. The suitable supplemental doses of TL106 were 5.0 × 10^8^ CFU/kg and 5.0 × 10^9^ CFU/kg.

### 5.4. Intestinal Morphology and SCFA Levels

**Figure 1:**
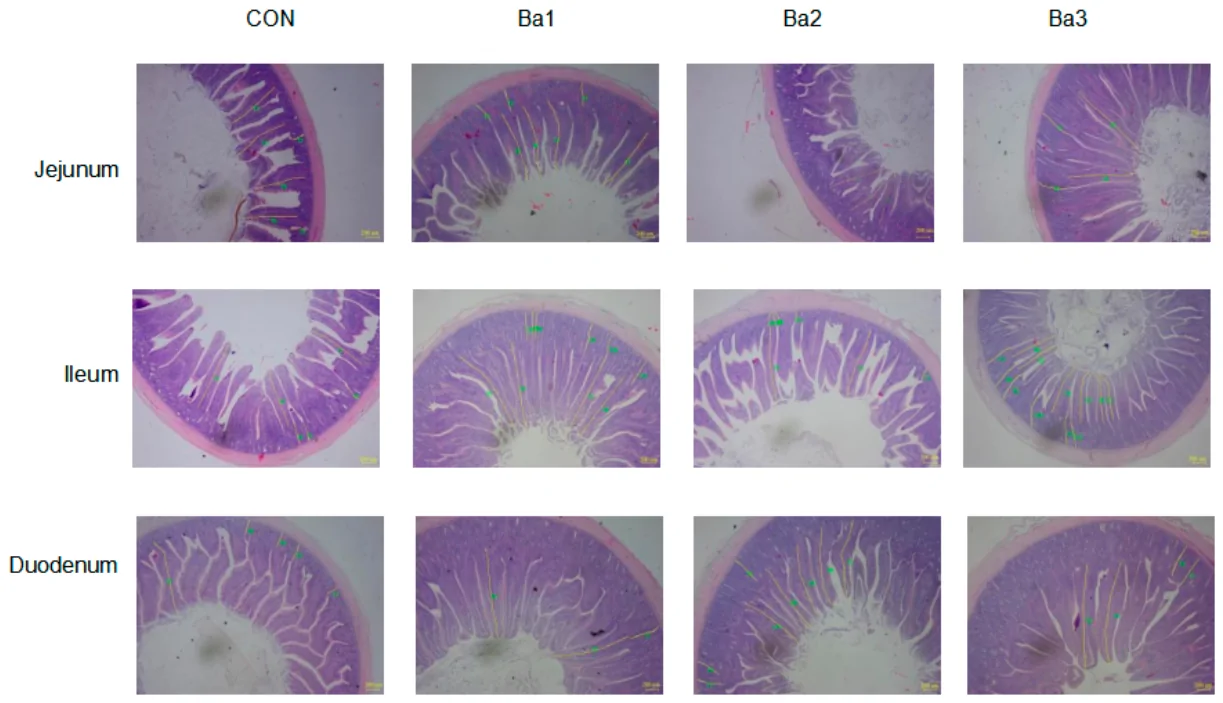
Effects of TL106 on intestinal morphology in Danzhou chickens at d 55.

The results are presented in Table 5 and Figure 1. At d 55, compared with CON, adding TL106 to diets had no significant effects on the morphology of the duodenum and ileum. However, the Ba3 group significantly increased VH/CD in the duodenum and ileum (*p* < 0.05), and both showed a quadratic curve relation (*p* < 0.05).

The results on SCFA levels in the cecal chyme are shown in Table 6. At d 35, butyric acid levels in Ba2 and Ba3 were significantly higher than those in CON and Ba1 (*p* < 0.05), and there was a quadratic curve relation (*p* < 0.05). There were hardly any differences in other SCFA levels among treatments. At d 55, butyric acid levels in the TL106 groups were significantly higher than those in CON (*p* < 0.01). The levels of propionic acid and valeric acid in Ba2 and Ba3 were significantly higher, and there were quadratic effects for butyric acid, propionic acid, and valeric acid (*p* < 0.05). Overall, adding TL106 had significant effects on butyric acid levels at two phases, particularly in the Ba2 and Ba3 groups. 

### 5.5. Cecal Microbiota Analysis

**Figure 2:**
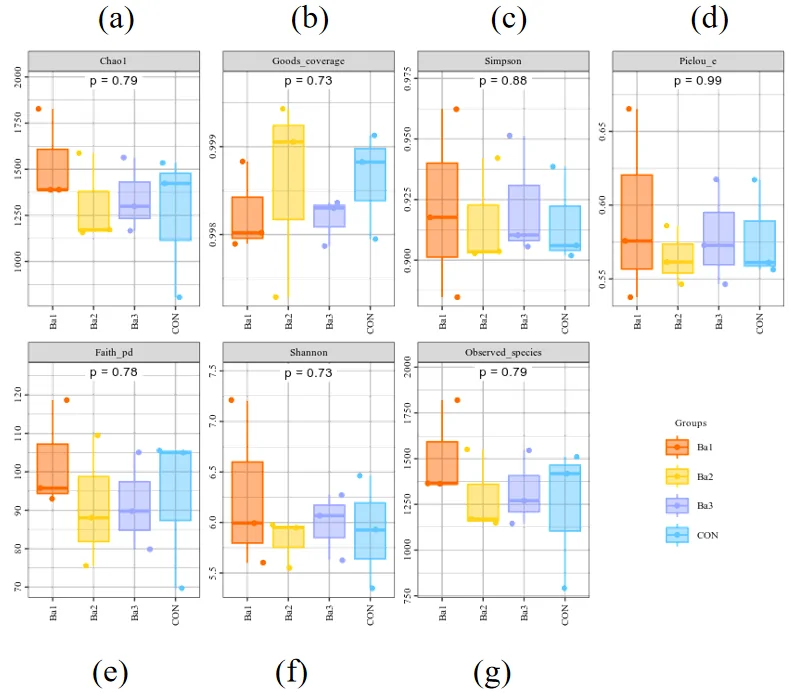
Boxplots of alpha diversity indices at d 35. Notes: (**a**) Box plot of the Chao1 index; (**b**) Grouped box plot of the Good’s coverage index; (**c**) Box plot of the Simpson index; (**d**) Grouped box plot of the Pielou’s evenness index; (**e**) Grouped box plot of the Faith’s PD index; (**f**) Grouped box plot of the Shannon index; (**g**) Grouped box plot of the observed species index. Using individual Danzhou chickens as the experimental units, *n* = 3 per treatment.

**Figure 3:**
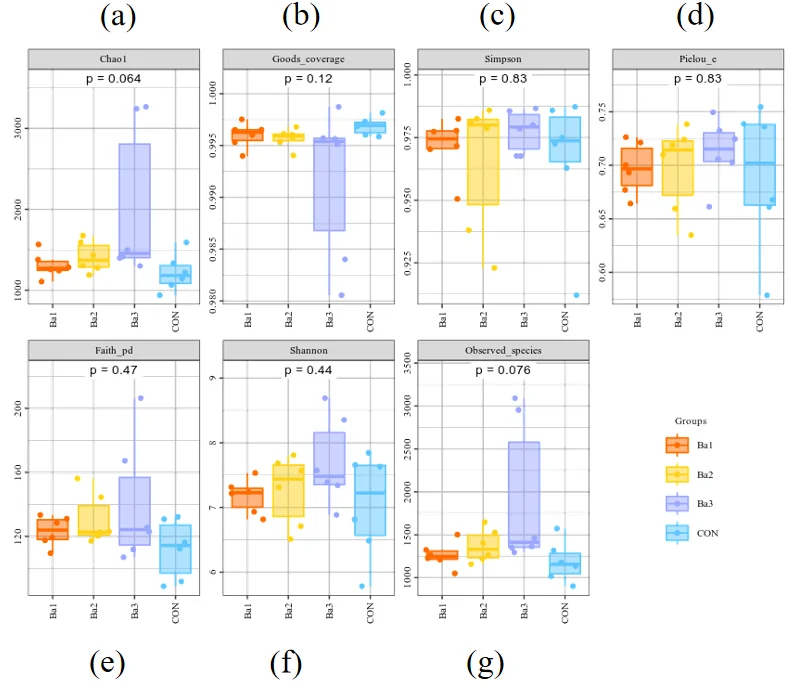
Box plots of alpha diversity indices at d 55. Notes: (**a**) Box plot of the Chao1 index; (**b**) Grouped box plot of the Good’s coverage index; (**c**) Box plot of the Simpson index; (**d**) Grouped box plot of the Pielou’s evenness index; (**e**) Grouped box plot of the Faith’s PD index; (**f**) Grouped box plot of the Shannon index; (**g**) Grouped box plot of the observed species index. Using individual Danzhou chickens as the experimental units, *n* = 6 per treatment.

Alpha diversity analy sis was employed to evaluate the microbial diversity within each group in Danzhou chickens (Figure 2 and Figure 3). At d 35, there were no significant differences in Chao1 and Observed species indices among groups (*p* > 0.05), indicating that TL106 addition had no effects on the richness of cecal microorganisms. At d 55, the differences tended to be significant (*p* < 0.1), suggesting that TL106 addition gradually increased the richness of cecal microorganisms. 

**Figure 4:**
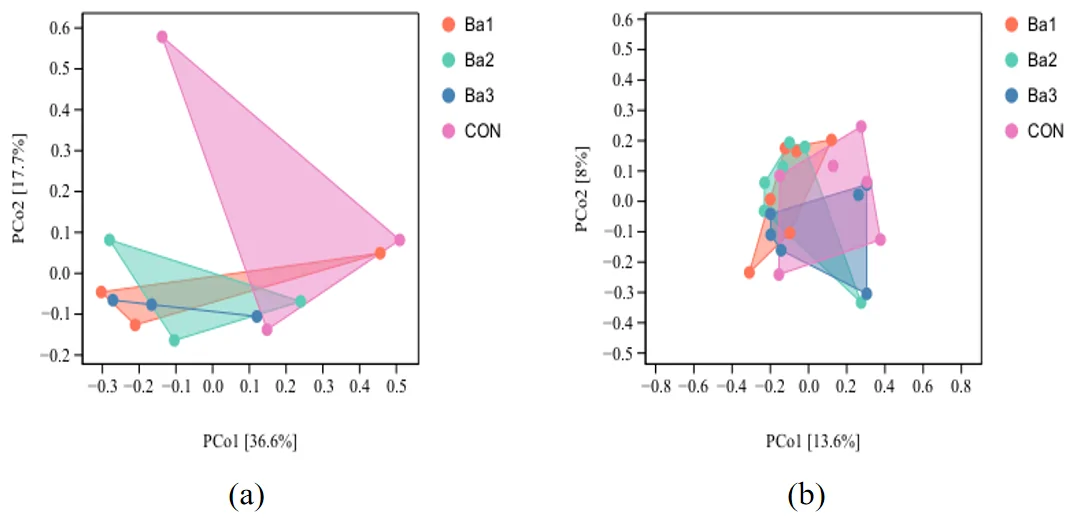
Principal coordinate analysis plots of cecal microbiota in Danzhou chickens. Notes: (**a**) principal coordinate analysis (PCoA) diagram of the cecal microbial community in Danzhou chickens at d 35. Using individual Danzhou chickens as the experimental units, *n* = 3 per treatment; (**b**) principal coordinate analysis (PCoA) diagram of the cecal microbial community in Danzhou chickens at d 55. Using individual Danzhou chickens as the experimental units, *n* = 6 per treatment.

The β-diversity index is used to reveal the differences in microflora composition. The analysis of cecal microbiota in Danzhou chickens was based on PCoA (Figure 4). At d 35, the sample points of each group were relatively dispersed. In particular, some points of CON were distant from those of TL106 groups on the PCo1 and PCo2 axes, without obvious overlapping aggregation. At d 35, TL106 groups exhibited a certain degree of differentiation in the microflora structure when compared with CON. At d 55, there was a greater number of overlapping sample points among the Ba1, Ba2, Ba3 and CON groups, with no discernible separation trend emerging between groups. This suggested a reduction in the differences in cecal microflora structure between the treated groups and CON, indicating that the influence of TL106 on β-diversity was weakened and the similarity of microflora structure increased. 

**Figure 5:**
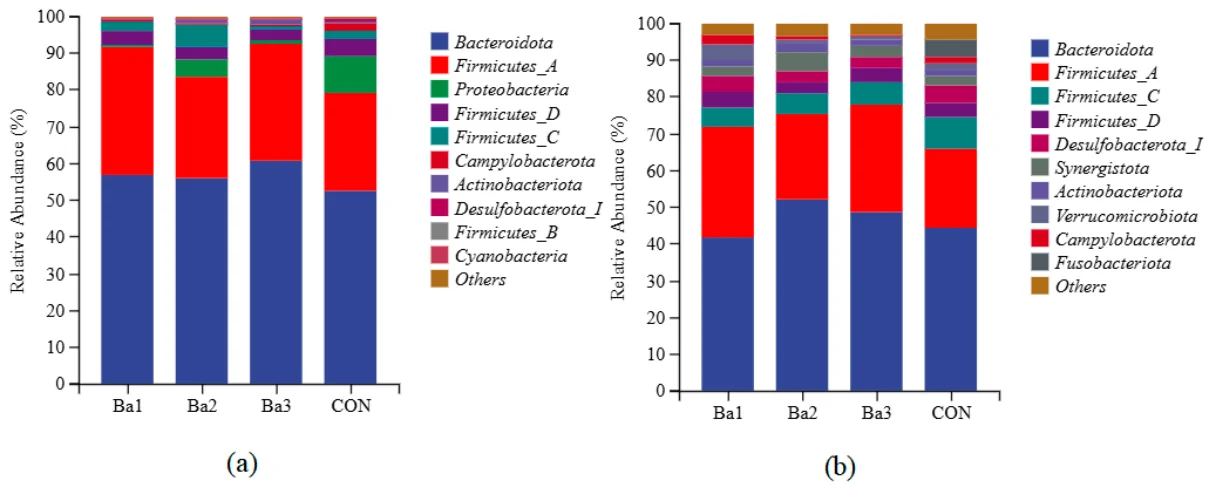
Plots of community abundance proportions at the phylum level. Notes: (**a**) Community abundance proportion at d 35 at the phylum level. Using individual Danzhou chickens as the experimental units, *n* = 3 per treatment; (**b**) Community abundance proportion at d 55 at the phylum level. Using individual Danzhou chickens as the experimental units, *n* = 6 per treatment.

The microbial community at the phylum level is shown in Figure 5. Bacteroidota and Firmicutes were the dominant species. At d 35, the top four communities of abundance in the TL106 groups were Bacteroidota, Firmicutes, Proteobacteria, and Campylobacterota. The abundances of Bacteroidota and Firmicutes in the TL106 groups were higher than in CON, and group Ba3 was the highest. Meanwhile, CON had the highest abundance of Proteobacteria. Desulfobacterota_I only existed in CON, but its abundance was low. At d 55, both Bacteroidota and Firmicutes were still the dominant phyla in the groups. The abundances of Bacteroidota in the Ba2 and Ba3 groups increased compared with those at d 35, while the abundances in CON and Ba1 decreased. Although the abundance of Firmicutes A decreased, its abundance in the TL106 groups was still higher than that in CON. Desulfobacterota appeared to be the most abundant in CON and Ba1. 

**Figure 6:**
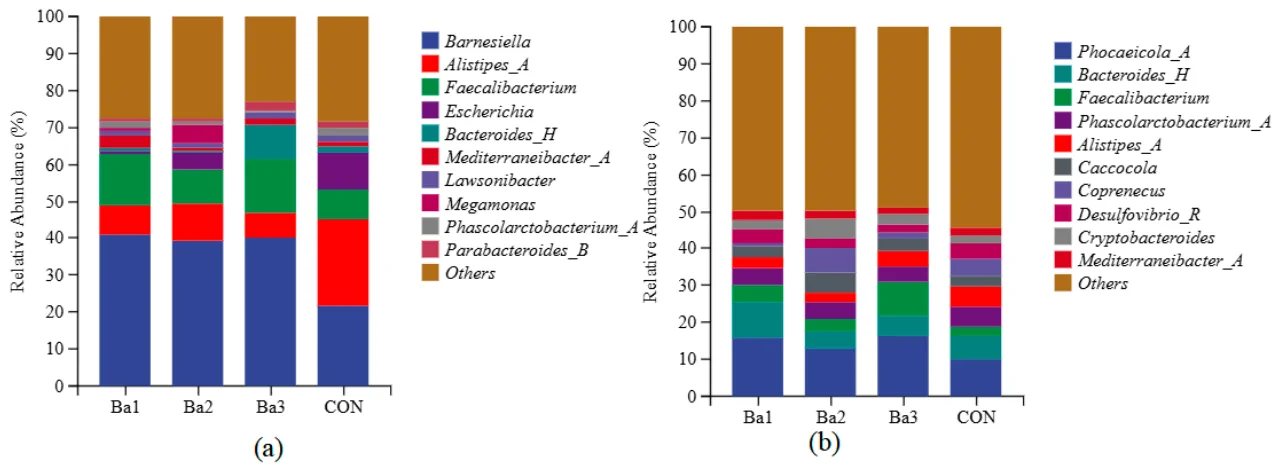
Plots of community abundance proportions at genus level. Notes: (**a**) Community abundance proportion at d 35 at genus level. Using individual Danzhou chickens as the experimental units, *n* = 3 per treatment; (**b**) Community abundance proportion at d 55 at genus level. Using individual Danzhou chickens as the experimental units, *n* = 6 per treatment.

Only the abundances of the top ten microbial genus communities are analyzed (Figure 6). At the early stage, the top five communities in groups were Barnesiella, Alistipes_A, Faecalibacterium, Escherichia, and Bacteroides_H. The abundances of Faecalibacterium in the TL106 groups were significantly higher than CON, and that of Alistipes_A was lower. The abundance of Bacteroides_H in Ba3 was higher than that in the others. At the later stage, the dominant genus had changed. Phocaeicola_A became the dominant genus, and the abundance of Bacteroides in CON was the lowest. The abundances of Faecalibacterium in TL106 treatments were all higher than that in CON.

**Figure 7:**
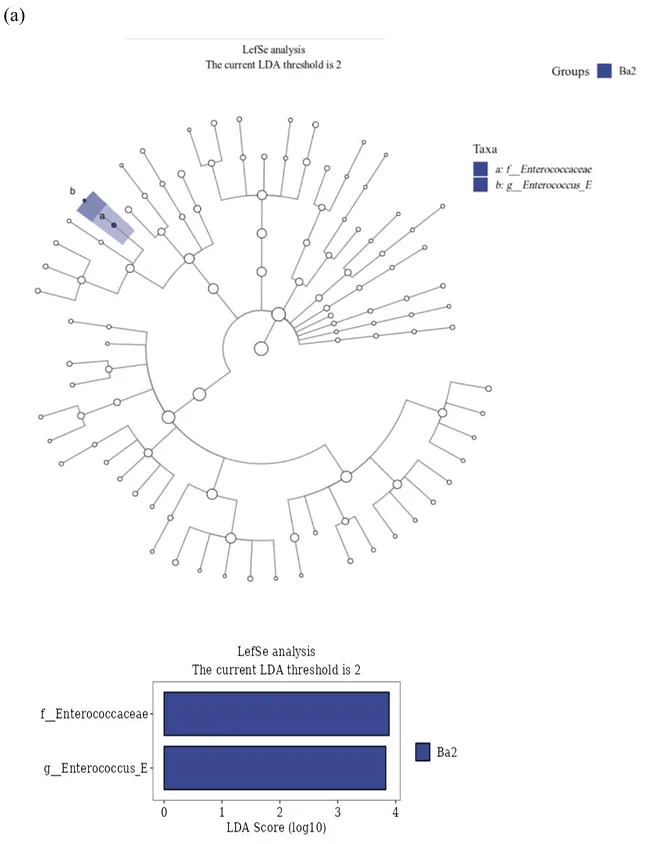

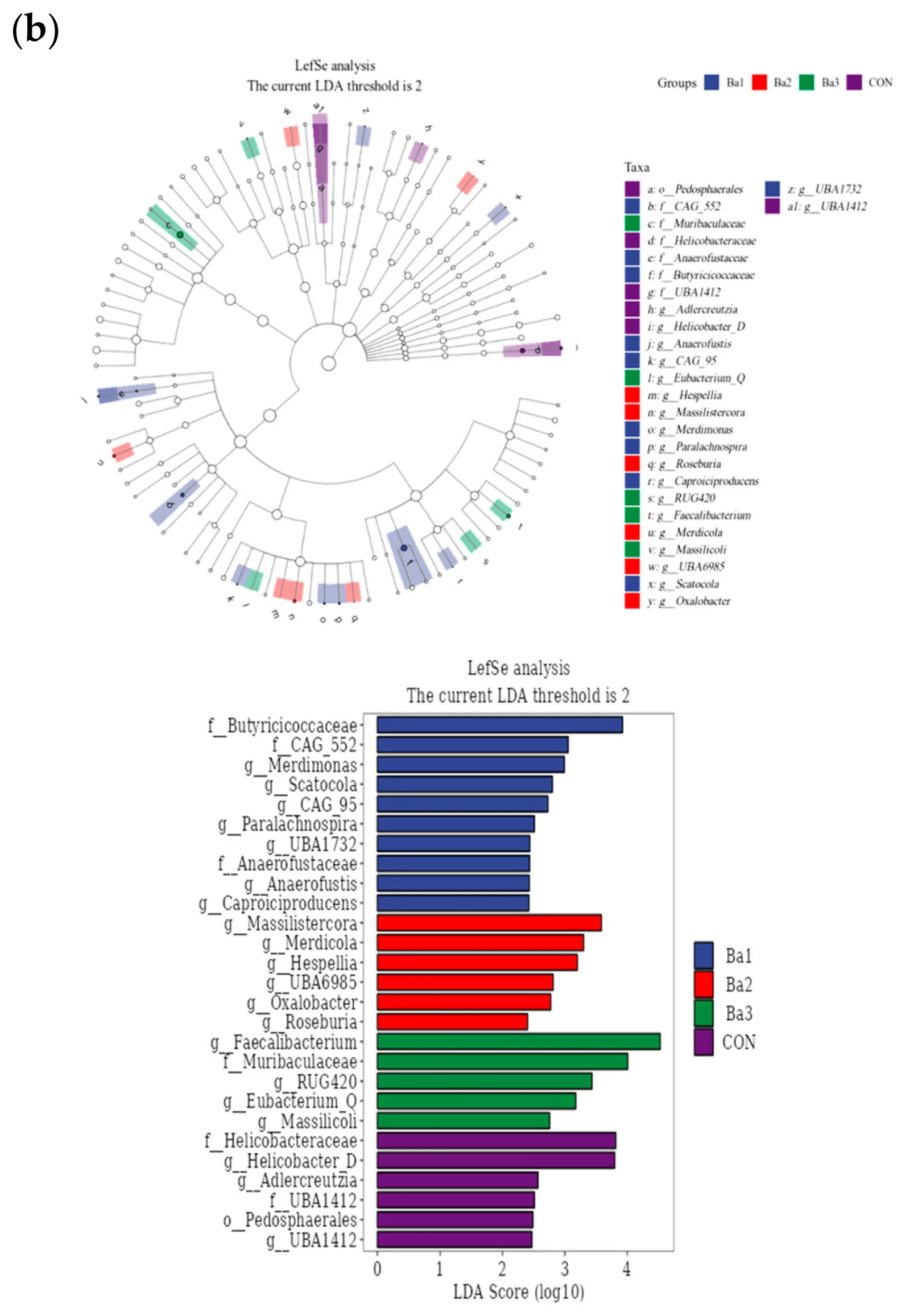
The LEfSe analysis for the marker species from phylum to genus. Note: (**a**) The LEfSe analysis of cecal flora from phylum to genus at d 35; (**b**) The LEfSe analysis of cecal flora from phylum to genus at d 55.

Figure 7 shows the differences in the microbial abundances of cecal chyme. For those with an LDA score of 2.0 or above, the effects of TL106 on the composition of cecal microbiota in Danzhou chickens were further elucidated by LEfSe analysis. At d 35, Enterococcus E in Ba2 was identified as the marker genus. At d 55, Merdimonas, Scatocola, CAG_95, Paralachnospira, UBA1732, Anaerofustis, and Caproiciproducens in Ba1 were the marker species. Massilicola, Merdicola, Hespellia, UBA6985, Oxalobacter, and Roseburia in Ba2 were the marker species. Faecalibacterium, RUG420, Eubacterium_Q, and Massilicolif in Ba3 were the marker species.

## 6. Discussion

Bllus species are extensively used as feed additives due to their beneficial effects on animal growth and maintenance of intestinal microbiota homeostasis. Previous studies have demonstrated that the inclusion of probiotics, such as Bacillus, in the diet can optimize growth performance of broilers [3,9]. This study showed that supplementation with TL106 during the late stage in Danzhou chickens significantly improved ADFI and reduced F/G. Bacillus strains can improve growth performance of poultry in the late stage [10], and a dosage threshold effect exists [11]. Long-term application of TL106 may indirectly promote nutrient absorption by regulating intestinal microbiota homeostasis and immune function [12]. The differences in probiotic effects across studies are closely related to strain characteristics, host breeds, and feeding environments [13].

Cytokines are key ind icators for evaluating the host’s immune status and inflammatory response. The dynamic balance of IL-6, TNF-α, and IL-10 is crucial for maintaining the intestinal barrier function [14]. This study found that the Ba3 group significantly inhibited the levels of IL-6 and TNF-α in Danzhou chickens at d 35, suggesting that TL106 may reduce pro-inflammatory cytokine levels, possibly through modulation of inflammatory signaling pathways, consistent with previous reports [15], potentially contributing to the regulation of early inflammatory responses. A study found that Swiss Lactobacillus KLDS1.8701 can balance T helper 17 cells and regulatory T cells (Treg), regulate IL-17 and TGF-β levels, and thus affect immune response [16]. At d 55, the Ba3 group significantly increased ALB and IgM content in Danzhou chickens, suggesting that TL106 may enhance antibody synthesis, consistent with an effect on humoral immune parameters. Moreover, the decreased TNF-α level was consistent with a potential anti-inflammatory effect. Bacillus TL106 may have similar functions by potentially inhibiting pro-inflammatory signals, for example, by secreting metabolites that block the TLR4/NF-κB pathway and reduce the synthesis of IL-6 and TNF-α [17]. TL106 may also regulate immune homeostasis by improving the microbial structure through the gut–immune axis [18]. The Ba3 group showed the best performance of Danzhou chickens in long-term use.

Antioxidant enzymes a nd oxidation products serve as indicators for assessing the body’s stress status. Five indicators, including T-AOC, T-SOD, CAT, GSH-Px and MDA, are commonly employed [19]. T-SOD alleviates oxidative damage by scavenging superoxide free radicals, and MDA, as the end product of lipid peroxidation, directly reflects the degree of oxidative stress [20]. Bacillus TL106 significantly enhanced the antioxidant capacity of Danzhou chicken. By 35 d, TL106 supplementation had elevated T-SOD activity and reduced MDA content. By 55 d, the Ba2 and Ba3 groups exhibited further decreased MDA levels. Concurrently, Ba2 demonstrated significantly higher GSH-Px activity, while Ba3 showed continued increases in T-SOD activity, both displaying quadratic relations. This indicated that TL106 may contribute to antioxidant status, possibly through modulation of enzymatic and non-enzymatic antioxidant systems [21], may be associated with improved glutathione-related antioxidant capacity [22], and may modulate gut microbiota [23]. The Ba3 group exhibited superior long-term effects, with dose- and time-dependent antioxidant effects.

Intestinal morphology correlates with intestinal health and function. VH, CD, and VH/CD directly reflect the proliferation and differentiation status of intestinal epithelial cells, as well as the nutrient absorption capacity [24]. An increase in VH and VH/CD can reflect the accelerated renewal of intestinal mucosal epithelial cells and a favorable differentiation state, ultimately enhancing digestive and absorptive functions [25]. The Ba3 group significantly improved VH/CD in the duodenum and ileum. The improvement was primarily attributed to a reduction in crypt depth, which is consistent with the mechanism through which probiotics enhanced the barrier function, rather than merely promoting villus elongation [23]. The quadratic relationship suggested a dose threshold effect, possibly related to the regulation of metabolites such as SCFA. Notably, although VH/CD in the jejunum did not display a significant change, its trend aligned with that of the duodenum and ileum, indicating potential functional synergy in the response to TL106 across different intestinal segments.

SCFAs serve as the pr imary metabolites of dietary fiber fermentation by intestinal microbiota. Butyric acid, propionic acid, and acetic acid play key roles in sustaining intestinal barrier function, modulating immune response, and facilitating energy metabolism [26]. The butyrate content in Ba3 at d 35 increased and was positively correlated with the increase in Enterobacter abundance. This trend continued to be higher than CON at d 55. This finding suggested that TL106 enhanced intestinal barrier function and anti-inflammatory effects by promoting the proliferation of butyrate-producing bacteria [27]. Butyrate, as a core metabolite, exhibited changes in concentration, which is consistent with the findings regarding SCFA in regulating intestinal function [28]. Moreover, the butyrate content decreased at d 55, which may be related to changes in the abundance of Enterobacter during the maturation of microflora.

TL106 addition regula ted the structure and function of cecal microbiota in Danzhou chickens, revealing the potential mechanism of probiotic effects through microbiota–host interaction. In terms of Alpha diversity, TL106 did not significantly improve microbial diversity at d 35. However, at d 55, there was a significant tendency in Chao1 and Observed indices, confirming its long-term beneficial effect on microbial richness. Similar findings were also reported in Ref. [6], which proposed that probiotics can inhibit pathogenic bacteria through competitive exclusion or by producing metabolic products, thereby fostering the proliferation of symbiotic bacteria. The PCoA analysis revealed significant differences in the dispersion of the sample points between TL106 groups and CON at d 35, but by d 55, all points began to overlap, suggesting that TL106 may have contributed to the maturation of the microflora, though this observation is exploratory and requires formal statistical validation [29].

At the phylum level, the abundance of Bacteroidota and Firmicutes in TL106 treatments was higher, while that of Proteobacteria decreased. Regarding the structure and function of microbiota, Bacillus DSM5749 has been reported to regulate intestinal microbiota and growth performance in broilers [30], which is consistent with these findings. Certainly, the ratio of Firmicutes to Bacteroidota (F/B) in intestinal microbiota significantly impacted growth performance in broilers [31]. A rising F/B in the cecum enhances carbohydrate metabolism efficiency, promotes volatile fatty acid production, and facilitates fat deposition through energy metabolism pathways [12]. At d 55, the elevated F/B in Ba3 resulted in a significantly higher ADG than that in CON. Additionally, a decrease in the abundance of Proteobacteria reduces the risk of potential pathogen infections [32]. Desulfobacterota only existed in CON, suggesting that the strain may reduce harmful metabolite accumulation by inhibiting sulfate-reducing bacteria [33].

At the genus level, t he abundance of Bacteroides and Bacteroides_H in the TL106 groups increased significantly, while that of Escherichia decreased [34]. The proliferation of Bacteroides may contribute to the increase in butyrate concentration, which can relieve intestinal inflammation by providing energy and likely by inhibiting the NF-κB pathway [35]. Furthermore, at d 55, Bacteroides (*Phocaeicola_A*) became the dominant genus in treated groups, suggesting that TL106 may maintain intestinal homeostasis by continuously regulating microbiota function [36].

## 7. Limitations

We acknowledge severa l limitations in this study. First, the mechanistic interpretations discussed above—including modulation of inflammatory signaling pathways, humoral immune activation, and gut microbiota–host interactions—are speculative and hypothesis-generating, rather than confirmatory, as they were not directly examined in this study. Second, the study was conducted in healthy Danzhou chickens without any inflammatory, oxidative, or pathogen challenge model, and the observed changes in serum parameters and cecal microbiota represent exploratory associations rather than direct causal evidence. Third, the 16S rRNA gene sequencing data describe relative compositional differences and do not establish microbial function, absolute abundance, or causal relationships linking microbiota changes to host physiological outcomes. Fourth, the identification of 5.0 × 10^8^ CFU/kg as the most effective dose among those tested is based on observed quadratic response patterns; rigorous determination of a global optimum would require formal dose–response modeling with a wider dose range and narrower intervals. Fifth, Duncan’s multiple-range test was used for post hoc comparisons, which provides relatively weak control of the family-wise Type I error rate; therefore, pairwise comparisons should be interpreted as exploratory. Future studies using challenge models, metabolomics, functional genomics, and more stringent multiple-comparison procedures are warranted to validate these findings.

## 8. Conclusions

In conclusion, dieta ry supplementation with *Bacillus amyloliquefaciens* TL106 at 5 × 10^8^ to 5 × 10^9^ CFU/kg was associated with improved growth performance (primarily during the later phase, 36–55 d), enhanced serum antioxidant enzyme activities, reduced lipid peroxidation, and modulation of selected immune parameters in Danzhou chickens under the conditions of this study. The cecal microbiota composition was altered, with an increased abundance of Faecalibacterium and elevated butyrate concentrations. However, all mechanistic interpretations remain speculative and were not directly tested. The findings should be interpreted as exploratory associations rather than causal evidence. Among the doses tested, 5.0 × 10^8^ CFU/kg appeared to be the most effective, but this should not be considered a statistically proven global optimum. Future studies using challenge models and targeted mechanistic approaches are needed to validate the proposed mechanisms and to establish causal relationships.

## Figures and Tables

**Table 1:** Composition and nutrient levels of the basal diet (as-fed basis, %).

Items	The Early Phase (1~35 d)	The Late Phase (36~55 d)
Ingredients (%)		
Corn	51.42	60.24
Cassava pieces	10.00	10.00
Dehulled soybean meal	26.00	20.00
Fermented soybean meal	8.00	6.00
Soybean oil	1.00	0.70
Limestone	1.15	0.90
Dicalcium phosphate	1.60	1.30
DL-methionine, 99%	0.11	0.12
L-lysine-HCL, 98%	0.02	0.04
Sodium chloride	0.30	0.30
Vitamin-mineral premix	0.40	0.40
Total	100.00	100.00
Calculated nutrient levels		
ME (Mcal/kg)	2.97	3.01
CP (%)	20.44	17.77
Calcium (%)	0.90	0.76
Available phosphorus (%)	0.42	0.35
Sodium chloride (%)	0.36	0.36
Total lysine (%)	1.15	0.98
Total methionine (%)	0.44	0.41

Notes: The premix provides the following per kg of diet: Vitamin A, 10,000 IU; Vitamin D3, 2750 IU; Vitamin E, 30 IU; Vitamin K3, 2 mg; Vitamin B1, 3 mg; Vitamin B2, 6 mg; Vitamin B6, 3 mg; Vitamin B12, 12 µg; nicotinic acid, 40 mg; D-pantothenic acid, 12 mg; biotin, 0.2 mg; choline chloride, 600 mg; iron (ferrous sulfate), 95 mg; copper (copper sulfate), 10 mg; manganese (manganese sulfate), 80 mg; zinc (zinc sulfate), 60 mg; selenium (sodium selenite), 0.30mg; iodine (potassium iodide), 0.35 mg.

**Table 2:** Effects of TL106 on growth performance in Danzhou chickens.

Items	Treatments				SEM	*p* Value		
	CON	Ba1	Ba2	Ba3		Interblock	Linear	Quadratic
Initial BW (g)	27.75	28.10	28.02	28.14	0.11	0.773	0.450	0.547
d 35 BW (g)	232.98	238.72	238.03	235.33	1.71	0.729	0.284	0.828
d 55 BW (g)	386.90 ^b^	417.88 ^a^	423.37 ^a^	424.01 ^a^	4.56	0.043	0.032	0.019
1–35 d								
ADG (g/d)	16.01	15.55	16.05	15.44	0.13	0.218	0.552	0.887
ADFI (g/d)	5.70	5.85	5.83	5.76	0.05	0.738	0.297	0.857
F/G	2.77	2.66	2.75	2.68	0.02	0.092	0.116	0.946
36–55 d								
ADG (g/d)	31.59	33.41	32.24	32.36	0.66	0.854	0.458	0.894
ADFI (g/d)	8.55 ^b^	9.95 ^a^	10.30 ^a^	10.49 ^a^	0.22	0.030	0.047	0.009
F/G	3.73 ^a^	3.37 ^ab^	3.14 ^b^	3.10 ^b^	0.08	0.031	0.128	0.005
1–55 d								
ADG (g/d)	21.20	21.51	21.45	21.08	0.24	0.918	0.628	0.869
ADFI (g/d)	6.65 ^b^	7.22 ^a^	7.32 ^a^	7.33 ^a^	0.08	0.045	0.034	0.019
F/G	3.19 ^a^	2.98 ^b^	2.93 ^b^	2.88 ^b^	0.04	0.061	0.107	0.018

Notes: Mean values within the same row with different superscript letters (a, b) are significantly different (*p* < 0.05). Using pen means as the experimental units, *n* = 6 pens per treatment, SEM = pooled SEM. Linear and quadratic *p*-values were derived from orthogonal polynomial contrasts using dose-level coding (0, 1, 2, 3 for 0, 5 × 10^7^, 5 × 10^8^, and 5 × 10^9^ CFU/kg). Abbreviations: CON represents Danzhou chickens fed on the basal diet; Ba1, Ba2, and Ba3 represent the addition of 5 × 10^7^, 5 × 10^8,^ and 5 × 10^9^ CFU/kg of TL106 in the basal diet, respectively.

**Table 3:** Effects of TL106 on cytokine and immunity indices in Danzhou chickens.

Items	Treatments				SEM	*p* Value		
	CON	Ba1	Ba2	Ba3		Interblock	Linear	Quadratic
d 35								
TP (g/L)	29.20	32.69	33.92	35.95	1.37	0.404	0.835	0.178
ALB (g/L)	16.91	17.31	17.92	18.29	0.38	0.633	0.391	0.989
IgA (mg/dL)	3.86	4.38	4.61	4.56	0.65	0.983	0.815	0.764
IgG (mg/dL)	3.02	4.85	3.99	5.85	0.68	0.555	0.555	0.502
IgM (mg/dL)	6.16	6.26	6.18	6.16	0.04	0.833	0.433	0.682
IL-6 (pg/mL)	109.01 ^a^	98.00 ^ab^	95.68 ^ab^	86.26 ^b^	3.10	0.043	0.272	0.023
IL-10 (pg/mL)	24.41	27.04	27.26	26.52	0.92	0.745	0.366	0.579
TNF-α (pg/mL)	95.34 ^a^	74.43 ^b^	76.21b	56.11 ^c^	4.53	0.001	0.047	0.002
d 55								
TP (g/L)	27.37	28.76	32.21	31.60	0.87	0.141	0.657	0.030
ALB (g/L)	10.32 ^b^	11.32 ^b^	12.90 ^a^	12.37 ^a^	0.37	0.045	0.379	0.012
IgA (mg/dL)	5.26	5.36	5.30	5.20	0.05	0.790	0.433	0.576
IgG (mg/dL)	4.99	5.06	5.14	4.88	0.06	0.507	0.139	0.895
IgM (mg/dL)	18.98 ^b^	18.27 ^b^	18.96 ^b^	19.27 ^a^	0.05	<0.001	0.001	0.002
IL-6 (pg/mL)	103.38	97.30	91.31	89.30	3.30	0.433	0.345	0.549
IL-10 (pg/mL)	18.03	19.40	21.66	24.04	1.31	0.416	0.144	0.483
TNF-α (pg/mL)	93.64 ^a^	78.62 ^ab^	64.28 ^bc^	59.77 ^c^	4.08	0.004	0.005	0.409

Notes: Mean values within the same row with different superscript letters (a, b, c) are significantly different (*p* < 0.05). Using individual Danzhou chickens as the experimental units, *n* = 6 per treatment; SEM = pooled SEM. Linear and quadratic *p*-values were derived from orthogonal polynomial contrasts using dose-level coding (0, 1, 2, 3 for 0, 5 × 10^7^, 5 × 10^8^, and 5 × 10^9^ CFU/kg). Abbreviations: TP, total protein; ALB, albumin; IgA, immunoglobulin A; IgG, immunoglobulin G; IgM, immunoglobulin M; IL-6, interleukin-6; IL-10, interleukin-10; TNF-α, tumor necrosis factor-alpha.

**Table 4:** Effects of TL106 on antioxidant capacity in Danzhou chickens.

Items	Treatments				SEM	*p* Value		
	CON	Ba1	Ba2	Ba3		Interblock	Linear	Quadratic
d 35								
GSH-Px (U/mL)	229.52	239.05	230.48	216.67	7.63	0.828	0.521	0.546
T-SOD (U/mL)	216.49 ^b^	345.01 ^a^	395.18 ^a^	428.74 ^a^	28.47	0.011	0.862	0.005
CAT (U/mL)	26.04	50.15	60.38	41.84	5.48	0.138	0.091	0.198
MDA (nmol/mL)	3.44 ^a^	2.69 ^ab^	2.37 ^b^	2.26 ^b^	0.18	0.049	0.011	0.253
T-AOC (mmol/L)	0.55	0.62	0.66	0.72	0.03	0.095	0.594	0.033
d 55								
GSH-Px (U/mL)	204.44 ^b^	240.00 ^ab^	313.70 ^a^	274.07 ^ab^	14.88	0.047	0.228	0.013
T-SOD (U/mL)	140.49 ^b^	137.28 ^b^	146.85 ^b^	157.88 ^a^	2.26	0.002	0.182	0.001
CAT (U/mL)	5.73	4.79	4.30	5.47	0.25	0.153	0.069	0.443
MDA (nmol/mL)	5.05 ^a^	5.03 ^a^	4.05 ^ab^	3.45 ^b^	0.22	0.013	0.745	0.002
T-AOC (mmol/L)	0.48	0.46	0.53	0.50	0.01	0.320	0.887	0.118

Notes: Mean values within the same row with different superscript letters (a, b) are significantly different (*p* < 0.05). Using individual Danzhou chickens as the experimental units, *n* = 6 per treatment; SEM = pooled SEM. Linear and quadratic *p*-values were derived from orthogonal polynomial contrasts using dose-level coding (0, 1, 2, 3 for 0, 5 × 10^7^, 5 × 10^8^, and 5 × 10^9^ CFU/kg). Abbreviations: GSH-Px, glutathione peroxidase; T-SOD, total superoxide dismutase; CAT, catalase; MDA, malondialdehyde; T-AOC, total antioxidant capacity.

**Table 5:** Effects of TL106 on intestinal morphology in Danzhou chickens at d 55.

Items	Treatments				SEM	*p* Value		
	CON	Ba1	Ba2	Ba3		Interblock	Linear	Quadratic
Duodenum								
VH (µm)	1086.02	1166.32	1195.63	1297.18	37.89	0.274	0.986	0.486
CD (µm)	477.77	456.97	450.20	356.35	21.46	0.176	0.711	0.052
VH/CD	2.28 ^b^	2.65 ^b^	2.74 ^b^	3.86 ^a^	0.19	0.010	0.928	0.002
Jejunum								
VH (µm)	877.68	944.21	1017.41	974.11	41.42	0.739	0.539	0.342
CD (µm)	412.12	404.64	388.66	308.79	19.45	0.194	0.734	0.126
VH/CD	2.27	2.41	2.69	3.23	0.17	0.171	0.934	0.065
Ileum								
VH (µm)	860.42	868.80	905.73	936.78	37.31	0.899	0.689	0.121
CD (µm)	336.22	301.28	215.87	198.07	27.75	0.239	0.87	0.133
VH/CD	2.95 ^c^	3.15 ^bc^	4.42 ^ab^	4.83 ^a^	0.27	0.019	0.993	0.014

Notes: Mean values within the same row with different superscript letters (a, b, c) are significantly different (*p* < 0.05). Using individual Danzhou chickens as the experimental units, *n* = 6 per treatment; SEM = pooled SEM. Linear and quadratic *p*-values were derived from orthogonal polynomial contrasts using dose-level coding (0, 1, 2, 3 for 0, 5 × 10^7^, 5 × 10^8^, and 5 × 10^9^ CFU/kg). Abbreviations: VH, villus height; CD, crypt depth; VH/CD, ratio of villus height to crypt depth.

**Table 6:** Effects of TL106 on SCFA levels of cecal chyme in Danzhou chickens (µg/g).

Items	Treatments				SEM	*p* Value		
	CON	Ba1	Ba2	Ba3		Interblock	Linear	Quadratic
d 35								
Butyric acid	398.36 ^b^	619.61 ^ab^	691.00 ^a^	729.05 ^a^	48.30	0.035	0.074	0.018
Acetic acid	2567.34	2827.71	2790.95	2860.13	152.53	0.930	0.660	0.723
Propionic acid	360.16	427.46	464.07	429.25	30.87	0.746	0.454	0.445
Valeric acid	77.10	82.30	89.38	86.37	6.22	0.933	0.777	0.581
Caproic acid	1.98	2.59	2.46	2.24	0.22	0.826	0.376	0.905
Isobutyric acid	53.48	39.07	43.67	50.39	2.86	0.298	0.072	0.892
Isovaleric acid	31.38	19.33	26.56	38.52	4.11	0.381	0.231	0.411
d 55								
Butyric acid	214.71 ^b^	403.86 ^a^	419.10 ^a^	495.93 ^a^	32.91	0.009	0.053	0.012
Acetic acid	1747.96	2163.05	2420.59	2644.19	134.04	0.093	0.364	0.029
Propionic acid	536.66 ^b^	641.62 ^ab^	802.69 ^a^	821.31 ^a^	42.27	0.038	0.380	0.007
Valeric acid	66.63 ^b^	90.53 ^ab^	128.31 ^a^	105.64 ^a^	7.64	0.022	0.119	0.008
Caproic acid	0.81	0.97	1.65	1.42	0.14	0.093	0.536	0.017
Isobutyric acid	87.85	90.84	125.21	87.75	7.39	0.211	0.473	0.241
Isovaleric acid	64.23	62.98	100.49	68.98	8.16	0.331	0.704	0.204

Notes: Mean values within the same row with different superscript letters (a, b) are significantly different (*p* < 0.05). Using individual Danzhou chickens as the experimental units, *n* = 6 per treatment; SEM = pooled SEM. Linear and quadratic *p*-values were derived from orthogonal polynomial contrasts using dose-level coding (0, 1, 2, 3 for 0, 5 × 10^7^, 5 × 10^8^, and 5 × 10^9^ CFU/kg).

## Data Availability

The data and models were not deposited in an official repository. The data from this study can be made available upon request from the corresponding authors.
